# OpenThread as a tool for localization of devices and patients in healthcare systems

**DOI:** 10.1007/s11548-026-03767-0

**Published:** 2026-08-04

**Authors:** Malte Voß, Vanessa Cobus, Frank Wallhoff, Armin Schneider

**Affiliations:** 1 Department of Engineering, Jade University of Applied Sciences, Wilhelmshaven, Germany; 2 Institute of Technical Assistance Systems, Jade University of Applied Sciences, Oldenburg, Germany

**Keywords:** Real-time location system, Workflow, Openthread, Bluetooth

## Abstract

**Purpose:**

Efficient patient flow management is a key factor for optimizing workflows in cost-intensive hospital units such as operating rooms. While existing prediction models can accurately estimate surgical durations, uncertainties in patient transport and availability of resources continue to affect daily schedules. Real-time location systems offer the potential to address this gap by providing information on patient and device locations. However, commercially available solutions are often costly and closed by providers. In this work, we present a scalable, research-oriented real-time location system, designed for low-power and low-cost deployment in hospital environments.

**Methods:**

Our system uses Bluetooth Low Energy beacons for device tracking and OpenThread as a transport layer to a database. A custom localization algorithm enables room-level assignment and triangulation-based localization in terms of coordinates. This setup is designed for low-power and low-cost deployment for research purposes.

**Results:**

Preliminary results demonstrate the feasibility of the proposed network for room-level assignment and position estimation of beacons while revealing notable variability in accuracy. Triangulation-based localization achieved a mean positioning error of 3.53 m, while room-level assignment showed error rates ranging from 0 to 40%, reflecting typical RSSI (Received Signal Strength Indicator) effects such as shadowing and signal reflections.

**Conclusions:**

The presented system provides an open, standards-based foundation for future improvements, including latency analysis, reliability assessments, advanced filtering methods and latest ranging techniques and represents a promising approach for adaptive patient flow management and clinical workflow optimization.

## Introduction

Operating rooms and examination rooms are the most cost-intensive units of any hospital, making it essential to optimize their workflow. To achieve this, various workgroups are developing prediction technologies to estimate operating time and to improve daily operation schedules [1–3]. These technologies use sensor data from medical devices, image analysis, and movements of instruments and personnel, to anticipate the intraoperative phase and predict the end time of the operation [4–6].

However, even with accurate operating time predictions, the start of the intervention remains an uncertain variable that can affect the daily schedule. Moreover, the prediction of anaesthesia introduction time is linked to several factors and becomes less precise with the difficulty of the case [7]. Another critical factor is the ideal time to transfer patients from the ward to the operating room, which depends on distance, obstacles, and resource availability.

Current research has focused on theoretical models of patient flows, such as Schmid and Doerner [8], which assessed the diagnostic and therapeutical route of a patient with respect to available resources and transport times. However, to transfer this theoretical intrahospital routing model into clinical practice, it is necessary to track patient locations and movements in real-time.

The benefits of precise patient planning and live localization are clear: reduced waiting times, improved patient satisfaction, and more efficient equipment utilization [9–12]

To achieve this, we propose a scalable research-centred Real-Time Location System (RTLS) based on OpenThread (OT) and Bluetooth Low Energy (BLE) to locate patients and devices in hospitals using low-power devices. While BLE-based localization and Thread-based mesh networks have been widely adopted in various applications, their combination to create RTLS in hospital settings is a novel approach. To the best of our knowledge, the presented approach is the first implementation of a RTLS that leverages the strengths of both BLE-based localization and Thread-based mesh networks to provide accurate and reliable location tracking of patients and devices in hospitals. This combination enables a decentralized and infrastructure-free RTLS that relies on open standards, low-power, and low-cost hardware, which makes the system particularly suitable for research applications.

Commercially available systems have a lack of access to all relevant data in real-time to calculate patient flows and to export necessary data due to interface incompatibilities. In addition, these systems are quite expensive compared to custom-made RTLS which overcome these disadvantages and offers a better scalability [13, 14].

## Material and methods

### Hardware and network infrastructure

The system is based on a BLE advertising infrastructure, in which advertisement packets are transmitted at a fixed interval of one packet per second. The advertisement payload includes a Universally Unique Identifier (UUID) corresponding to the Bluetooth Indoor Positioning Service (IPS), which allows the receiving infrastructure to selectively identify relevant packets and ignore non-related BLE traffic [15]. For BLE beacons, the Nordic Thingy:53 (Nordic Semiconductor, Trondheim, Norway) is used, as these are already equipped with a battery and are programmable from the ground up. As programming tools for the nRF beacons, the Connect SDK v2.8.0 and the Toolchain v2.8.0 were used.

To receive BLE advertisement packets, OT-enabled nodes are deployed as stationary receiver anchors with known coordinates. OT provides a complete, standards-compliant Thread stack that can run on resource-constrained microcontrollers. The network operates in the 2.4 GHz ISM band and implements IPv6 over Low-Power Wireless Personal Area Networks (6LoWPAN) for efficient packet compression. OT nodes were realized with a wide range of microcontrollers, such as the nRF52840 Dongle, nRF52840 DK, nRF54L15 DK (all Nordic Semiconductor, Trondheim, Norway), Xiao nRF52840, Xiao nRF54L15 (Seeed Technology, Shenzhen, China), Arduino Nano 33 BLE Sense (Arduino, Somerville, MA, USA) and nice!nano board (Nice Technologies, Fort Wayne, IN, USA). This diverse hardware approach was adopted to assess the integration capabilities of available commercial devices. Furthermore, the variety of platforms facilitates a comparative evaluation of power consumption characteristics, supporting the long-term objective of autonomous OT-node operation for scientific experiments. All selected platforms are based on either nRF52840 or nRF54L15 System-on-Chip (SoC) which supports both BLE and OT, enabling the concurrent operation of BLE scanning and OT networking on a single device. For programming, the nRF Connect SDK v3.1.1 and Toolchain v3.1.1 were used.

To achieve communication between the OT Network and the LAN, a Raspberry Pi 3B+ with an nRF52840 dongle acts as an OT Border Router to bridge the OT network to the LAN. Therefore, the official OTBR Docker image and an nRF52850 Dongle running the official Co-processor firmware provided by Nordic Semiconductor were used [16].

### Message transmission

**Fig. 1:**
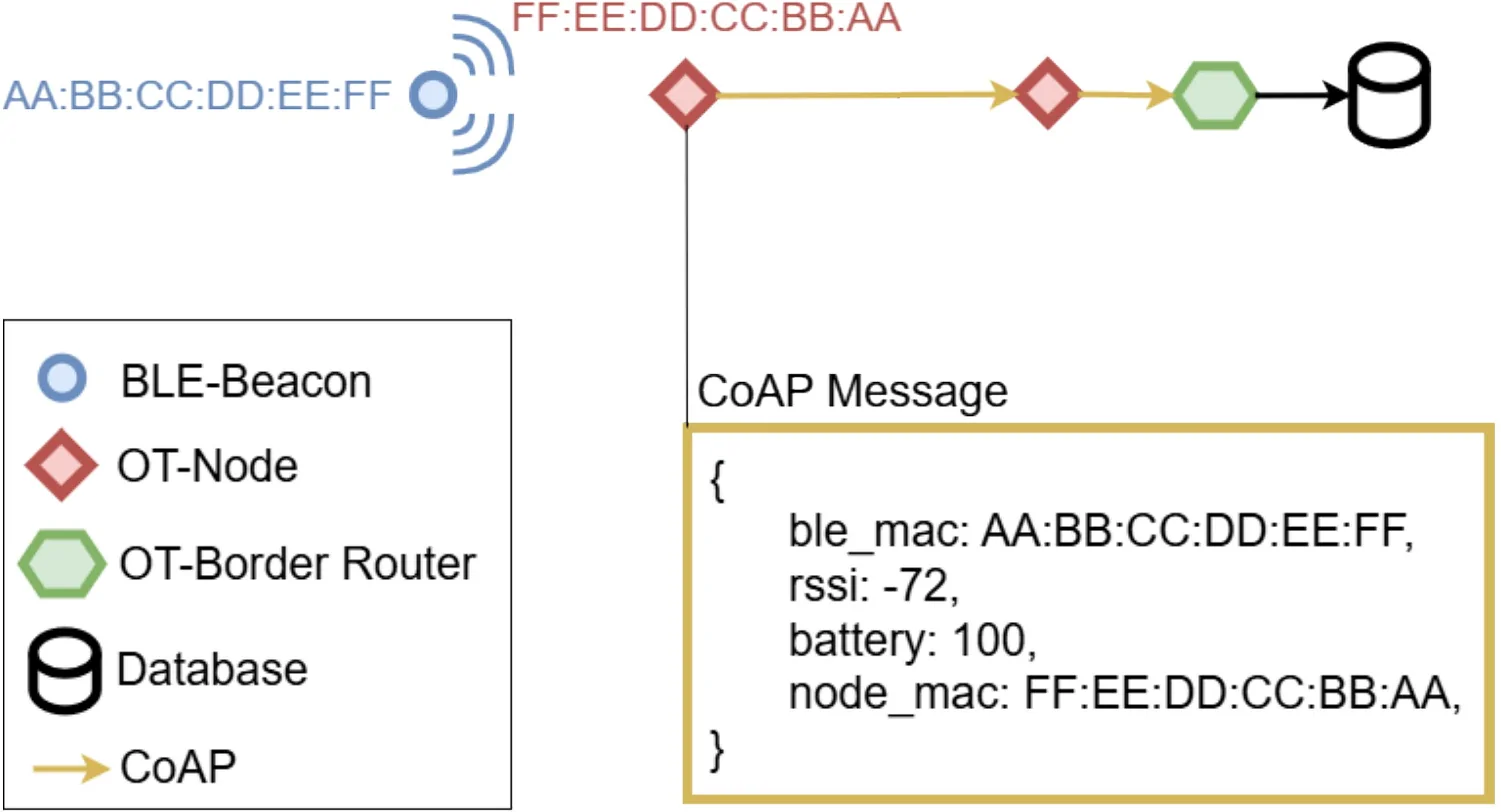
Process in which the message from the BLE beacon's is transmitted via the OT network and stored in a database. OT-enabled device extracts the MAC address, RSSI value, and battery level from the BLE beacon and appends its own MAC address, before transmitting the data to a CoAP server via CoAP and storing it in a database

Each node continuously scans for BLE advertisement packets from beacons containing the IPS UUID. Upon reception of a packet, the node extracts the beacons MAC address, the measured received signal strength indicator value (RSSI) and the battery level. To identify the receiver, which captured the packet, the MAC address of the node is appended before transmitting the message to the server. Each OT-Node is configured as a full Thread device, and each OT-Node transmits the message via the Constrained Application Protocol (CoAP) to a centralized server independently of other OT-Nodes. Figure 1 shows a schematical overview of the described process.

### Processing and position calculation

At server side, incoming CoAP messages are processed and stored in a PostgreSQL database. Each record is augmented with a server-side timestamp to differentiate between advertisement packets coming from the same beacon. The database represents a time-resolved collection of RSSI measurements, indexed by beacon identity and receiver location.

To locate the beacons, we developed a custom algorithm that combines RSSI-based distance estimation with triangulation and Kalman filtering. The algorithm consists of the following main steps:

1. RSSI-based distance estimation: For each beacon, the algorithm calculates the distance to each OT-Node based on the received RSSI values. We use a standard path loss model to estimate the distance, which is then stored in the database.
2. Triangulation: Using the distances calculated in the previous step, we apply a triangulation algorithm to estimate the position of the beacon. The algorithm uses the positions of the OT nodes and the estimated distances to calculate the coordinates of the beacon.
3. Filtering: Instead of a full probabilistic Kalman filter, a weighted moving average over the most recent position estimates is used. The filter maintains a history of the last five position estimates per beacon. The applied weights increase linearly for more recent observations, emphasizing newer measurements. Specifically, for a maximum history size of five, the weights are defined as ([0.1, 0.15, 0.2, 0.25, 0.3]), with the most recent position receiving the highest weight. If fewer than five samples are available, the corresponding subset of weights is used.

In addition to coordinate-based localization, we implemented a secondary room-level assignment mechanism to address the limitation of occasional inconsistent estimates near walls, doors, or areas with strong signal reflections. The mechanism performs a voting-based room classification using the RSSI measurements directly. For each received advertisement packet, all OT nodes that detect the packet participate in a voting process, contributing a weighted vote for associated room based on the measured RSSI value. Stronger signal strengths result in higher voting weights, reflecting closer proximity between the beacon and the receiver. The room with the highest aggregated vote is selected as the beacon’s current room assignment, ensuring that the beacon is consistently associated with a plausible room.

## Test setup

**Fig. 2:**
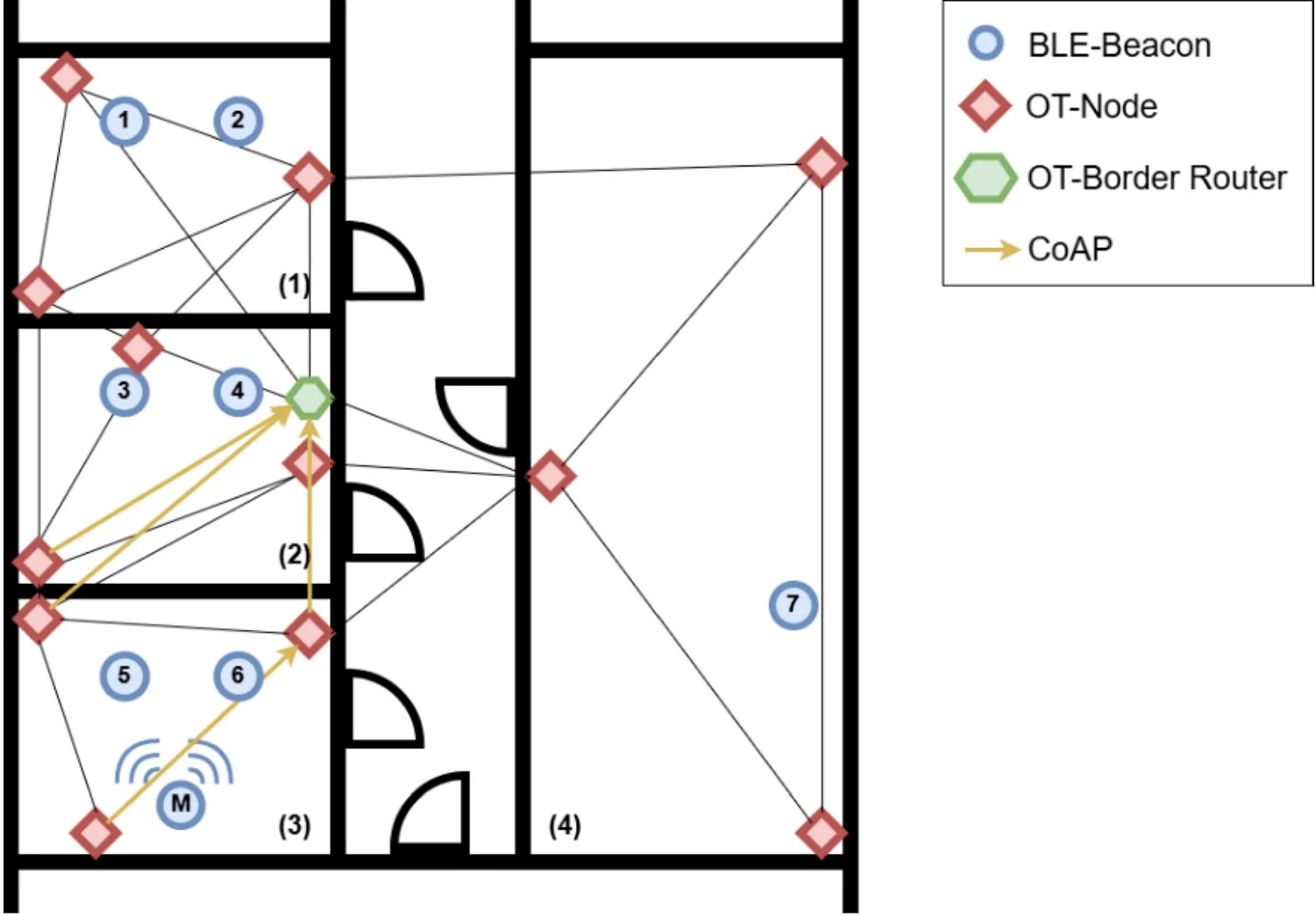
Beacons (1…M) are detected by multiple OT-Nodes, which forward RSSI measurements via CoAP to an OT border router for centralized processing and position estimation. (1) room with medical equipment, (2) patients’ room, (3) nurses’ station, (4) delivery room

The current implementation of the proposed RTLS is evaluated in a laboratory environment at the Jade University of Applied Sciences. The test area consists of four adjacent rooms, each equipped with three OT nodes acting as receivers for the BLE packets (see Fig. 2).

The rooms are set up to simulate different areas of a hospital, with one room configured as nurses’ station, one room representing a patients’ room, one room equipped with medical equipment such as heated beds, and the fourth room designed to mimic a delivery room. In three of the four rooms, two stationary beacons are permanently placed to provide reference signals, while the fourth room contains only one beacon, to mimic realistic conditions of a hospital ward with patient rooms. An additional mobile beacon is placed in a randomized manner across the rooms. For each trial, the time of entering a room is recorded, then the beacon remains stationary for approximately one minute. Afterwards, the time of leaving the room is recorded. Each of the four rooms was visited 15 times in a randomized order, resulting in a total of 60 trials. This experimental design primarily aims to assess the reliability of room-level assignment of beacons under realistic hospital conditions.

**Fig. 3:**
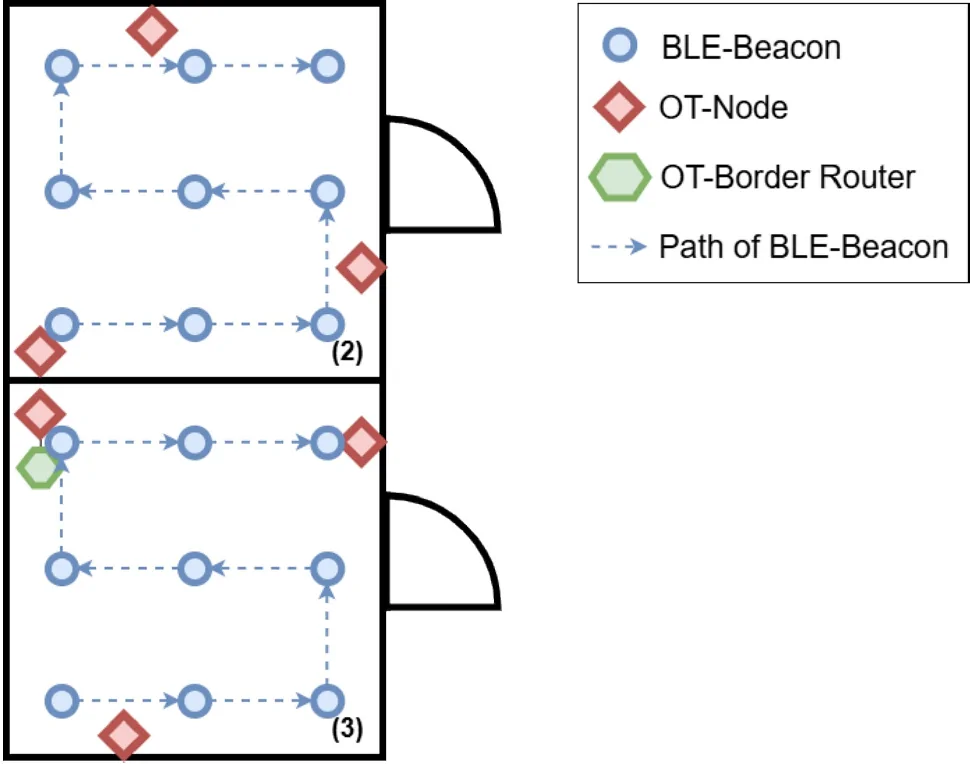
Path the BLE-Beacon was moved along to evaluate position errors. (2) patients’ room, (3) nurses’ station

A second test was performed to address the triangulation-based method. Therefore, two rooms were equipped with three OT nodes each. One beacon was then moved through the rooms at nine fixed positions per room (see Fig. 3), also remaining stationary for one minute at each position.

## Results

**Fig. 4:**
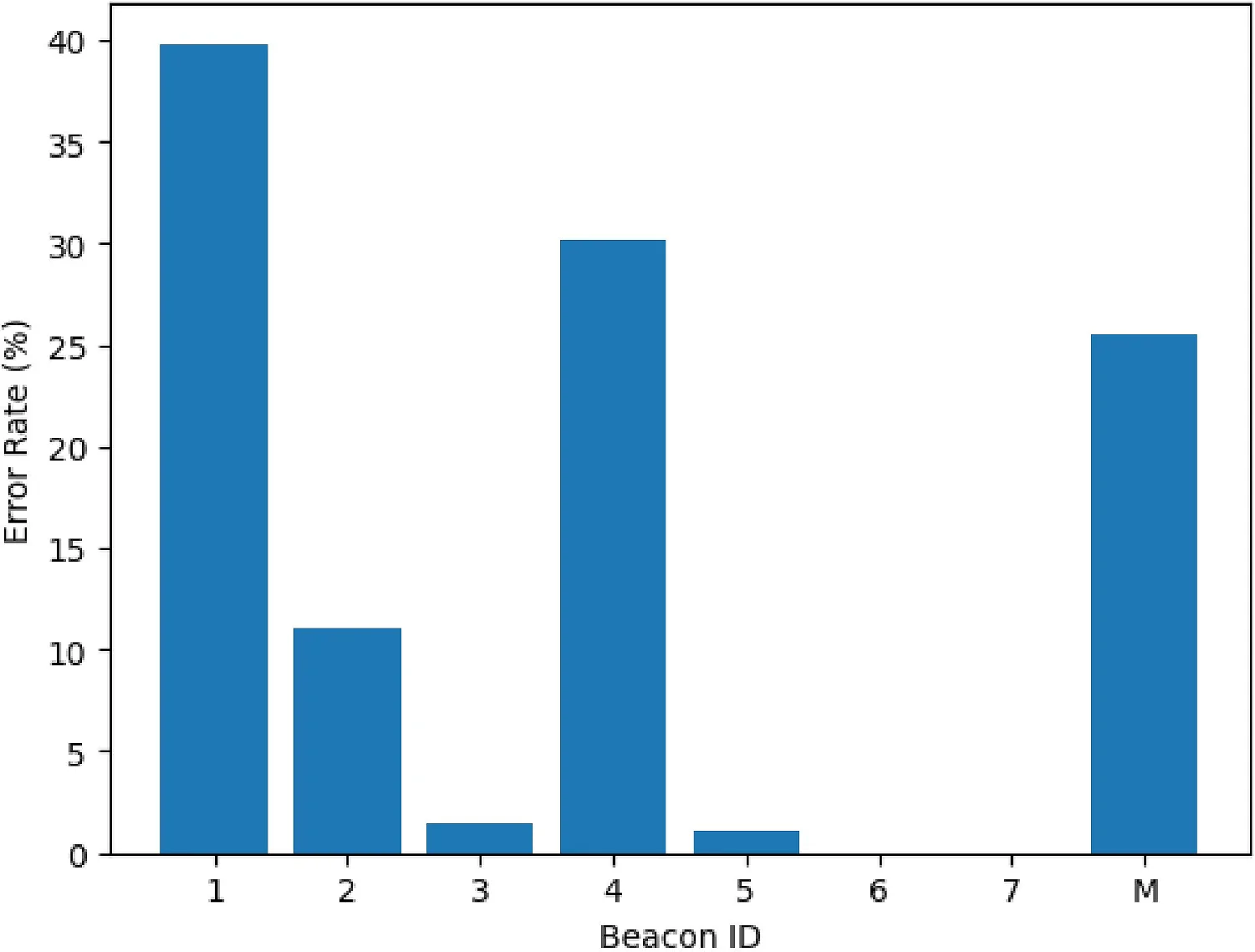
Error rates across beacons on mismatches between expected and assigned room number. The moving BLE beacon M was randomly moved across all rooms

For evaluating the reliability of room-level beacon assignment, an error rate was calculated for each individual beacon as well as for each room, based on mismatches between the expected and the assigned room labels. While having beacons exhibited very low error rates ranging from 0% to 1.5% (e.g. beacons 6, 7, and 5), others showed substantially higher error rates of up to 40% (beacon 1), indicating pronounced variability in room assignment performance (Fig. 4).

**Fig. 5:**
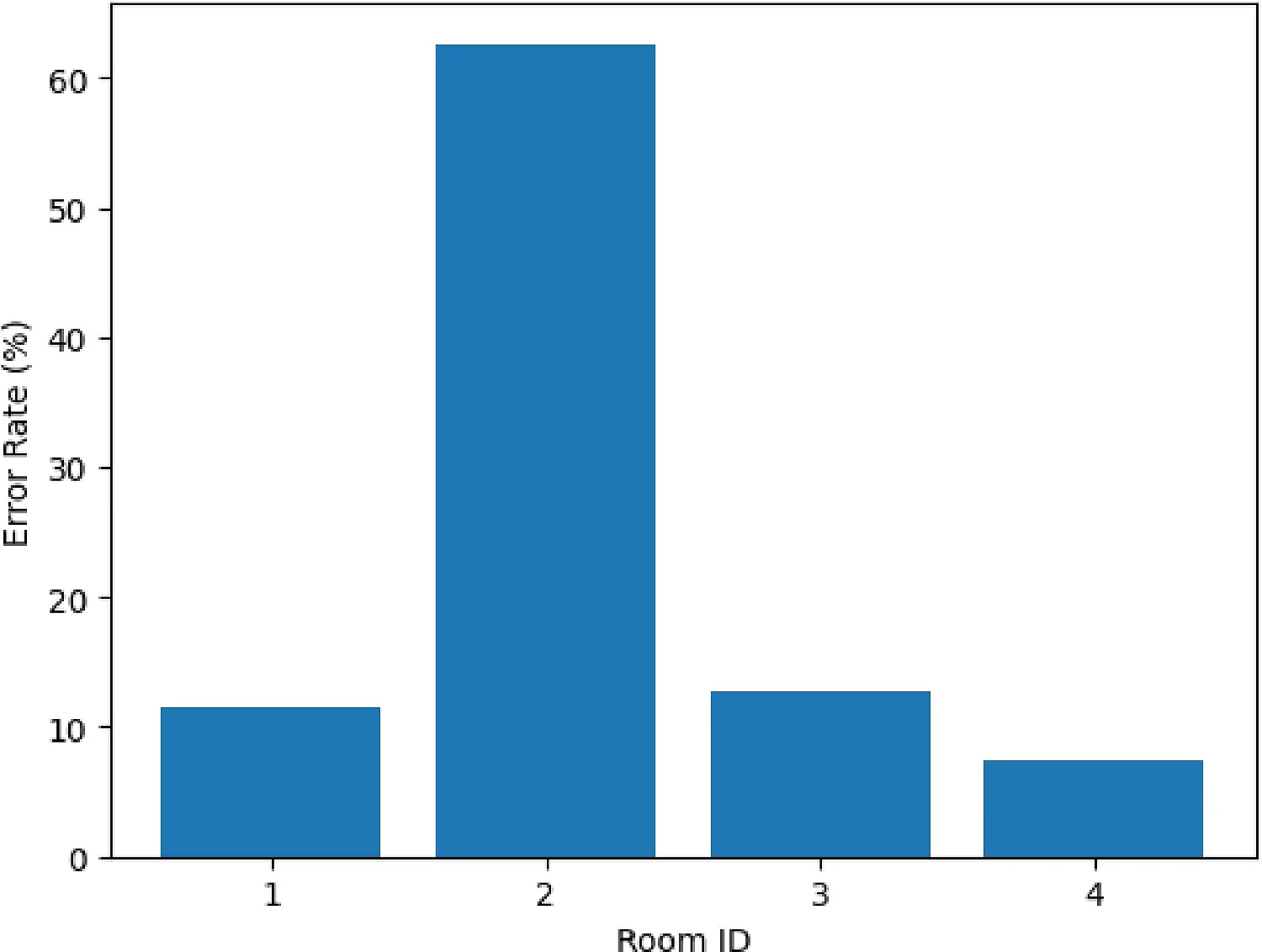
Error rates across rooms based on mismatches between expected and assigned room id

Figure 5 shows the resulting error rates in different rooms. While the current assignment accuracy does not yet match clinical requirements, the observed error patterns in different rooms show, that further experiments to assess room and microcontroller variability is necessary. This became particularly evident by completely different error rates in two nearly similar equipped rooms.

**Fig. 6:**
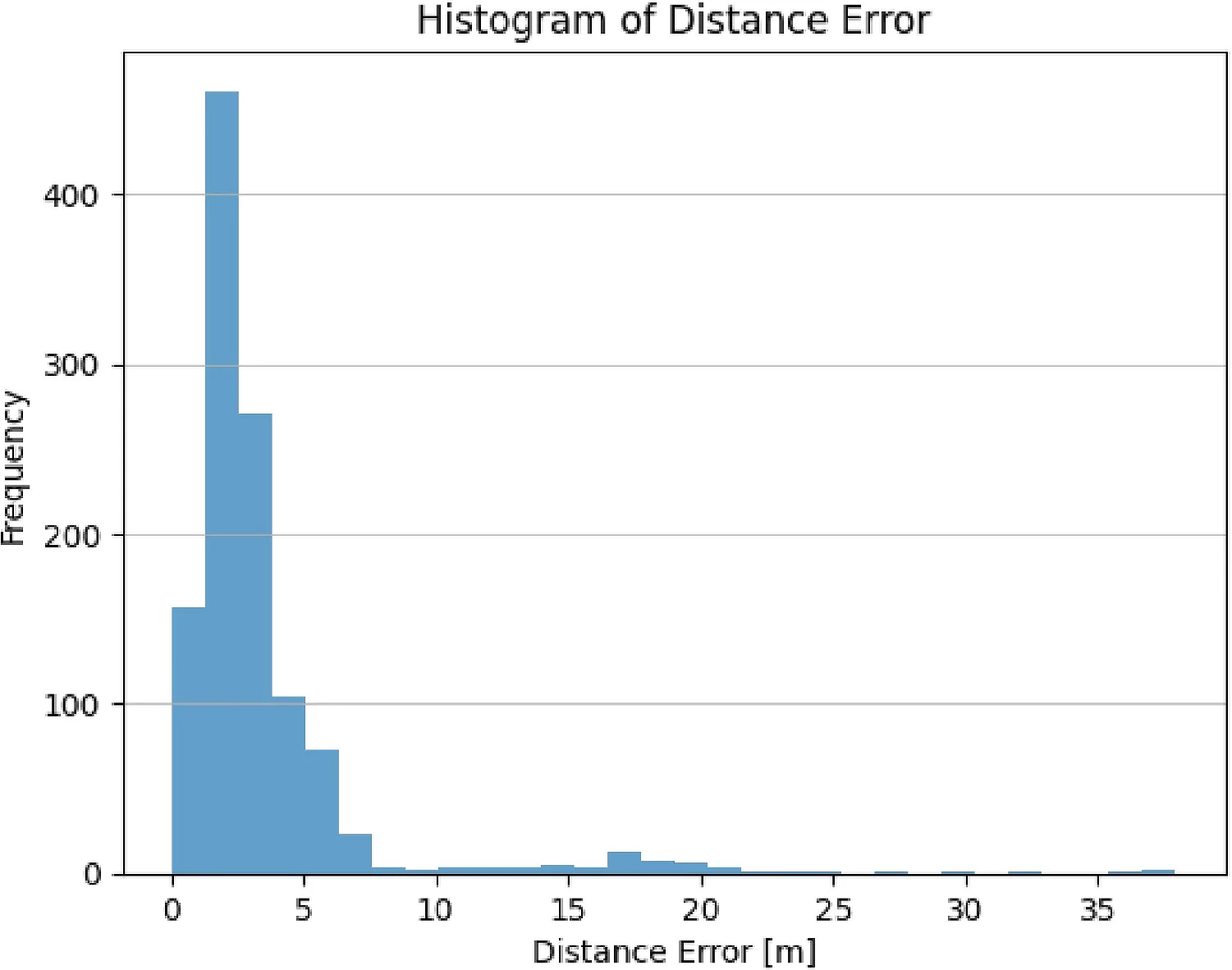
Histogram shows the frequency of distance errors occurred in assessments of the triangulation-based method

Calculation of the position error in the triangulation-based method was done through the Euclidian distance for each advertisement interval per beacon. In total the system showed a mean error of 3.53 m (± 4.00 m SD). Figure 6 shows the histogram of distance errors.

## Discussion and future work

The preliminary results demonstrate the feasibility of an OT-based RTLS for room-level and triangulation-based localization system under realistic indoor conditions. Typical characteristics of RSSI measurements, such as signal reflection or shadowing remain key challenges for accurate position estimation and room assignment. Although a non-negligible distance error persists, careful selection of node placement enables the extraction of a patient bed’s location within the corresponding room (e.g. operating room, preparation room, waiting room) from the collected data. These findings, however, confirm the technical feasibility of the system and highlight the need for advanced filtering, aggregation and position algorithms. Additionally, a current limitation concerns timestamps. These are applied only at server level. While OT specification generally supports Simple Network Time Protocol (SNTP) for distributed synchronization, the radio driver provided in the Nordic Connect SDK does not support the feature [17]. This constraint prevents node-level timestamping and must be considered when analysing system latency.

Future work will focus on a systematic analysis of system latency and its impact on room assignment performance, as well as on evaluating the end-to-end reliability of the communication chain. This includes assessing whether all BLE advertisement packets received by the OT nodes are consistently forwarded to and processed by the backend server. Additionally, advanced aggregation and filtering strategies will be investigated to improve robustness and clinical applicability. Since in our system setup a mix of different microcontrollers acting as nodes is used, we will also focus in our future evaluations on differences in RSSI measurements between the hardware devices.

Future work will also investigate on latest mechanisms for distance measurements such as Bluetooth Channel Sounding to overcome the typical RSSI challenges by introducing round-trip time and phase-based ranging.

In the long term, the proposed system is intended to support adaptive patient flow management by providing reliable real-time location data that can be directly integrated into clinical workflow optimization approaches.

## Conclusion

This work presents a scalable, research-oriented RTLS design, based on OT and BLE, aiming to enable real-time and live localization of patients and devices in hospital environments using low-cost, low-power hardware. Thread was selected for its low-power consumption, IPv6 connectivity, self-healing topology, and native support for constrained devices. The laboratory evaluation demonstrates the technical feasibility of both room-level assignments and triangulation-based position calculations under realistic indoor conditions, while also revealing current limitations in accuracy caused by typical RSSI effects. Despite these limitations, the system architecture provides a flexible and standards-based foundation for future improvements. The presented results support the potential of OT-based RTLS as a viable alternative to proprietary solutions and motivate further research towards latency-aware, reliable, and clinically applicable localization system. Currently Nordic Semiconductor-based hardware is implemented; however, as the system is based on open and standard protocols, alternative widespread used hardware platforms, such as Espressif’s ESP32 series, are in principle also applicable, if support for BLE and OT networking is simultaneously possible.

## Data Availability

The source code developed for this study is still under active development and is undergoing further validation. The authors commit to make the code publicly available in a GitHub repository upon completion of the validation process.
